# Integrating Intrinsic Mechanisms, Environmental Drivers and Anthropogenic Influences in Long-Lived Tree Longevity

**DOI:** 10.3390/plants15111638

**Published:** 2026-05-27

**Authors:** Zhangxiang Min, Yifeng Chen, Zhiwei Chen, Bingsong Zheng, Daoliang Yan

**Affiliations:** 1 State Key Laboratory for Development and Utilization of Forest Food Resources, Zhejiang A&F University, Hangzhou 311300, China; 2 Hangzhou Woodpecker Ancient Tree Rescue Co., Ltd., Hangzhou 311300, China

**Keywords:** long-lived trees, intrinsic mechanisms, environmental drivers, anthropogenic influences

## Abstract

Long-lived trees represent a remarkable biological phenomenon characterized by exceptional lifespan, ecological resilience, and cultural significance. However, current studies on plant longevity have primarily focused on intrinsic physiological and genetic mechanisms, while the integrated roles of environmental and anthropogenic factors remain insufficiently explored. This review synthesizes current knowledge on tree longevity from an integrative perspective. We revisit the relationship between senescence and longevity, emphasizing that plant aging is not merely functional decline but a regulated and adaptive process. We further summarize intrinsic mechanisms associated with longevity, including nutrient allocation, antioxidant defense, hormonal regulation, secondary metabolism, and molecular maintenance. In addition, we highlight the importance of environmental drivers and anthropogenic influences in shaping long-term survival and stability of long-lived trees. Finally, we propose a four-dimensional coupling framework integrating resource allocation, stress resistance, environmental pressure, and anthropogenic regulation. Within this framework, tree longevity is interpreted as an emergent property maintained through dynamic interactions, resilience, and feedback regulation. This review provides a conceptual basis for understanding plant longevity and offers theoretical support for the conservation of long-lived trees under global environmental change.

## 1. Introduction

Long-lived trees constitute a remarkable and irreplaceable group within the plant kingdom, characterized by lifespans extending from several centuries to over a millennium. They embody the cumulative outcomes of long-term evolutionary selection and environmental adaptation, representing a unique biological and ecological phenomenon. As witnesses to both natural history and human civilization, long-lived trees possess extraordinary historical, cultural, economic, and aesthetic significance. Beyond their cultural symbolism, they serve as invaluable genetic reservoirs and ecological archives, retaining information on past climatic, hydrological, and vegetational changes that are essential for reconstructing environmental history [1]. Their ability to persist through centuries of ecological competition and disturbance also makes them keystone representatives of regional biodiversity and ecosystem stability.

In Western countries, systematic research on long-lived trees began relatively early, with conservation-oriented studies emerging as early as the 1940s. Early investigations primarily focused on the causes of senescence, physiological aging processes, and the ecological value of old-growth individuals [2]. In the United States and Germany, classification criteria extend beyond chronological age to include rarity, morphological distinctiveness, and historical relevance. In the United Kingdom, the term Historic Trees refers to trees of exceptional ecological, cultural, or historical value. Such trees are recognized for attributes including advanced age, provision of critical wildlife habitats, exceptional morphological characteristics relative to conspecifics, associations with major historical events, or outstanding cultural significance [3]. Singapore, as a relatively young nation, does not categorize trees strictly by age. Instead, it employs a broader designation—the Heritage Tree. Comparative analysis indicates that Singapore’s definition is more inclusive, emphasizing ecological, cultural, and historical values and positioning tree conservation as an integral component of national heritage protection [4].

In China, scientific research and conservation practices began later but evolved rapidly through a distinct national framework driven by policy innovation. The earliest official definition, introduced in 1963, emphasized commemorative significance, rarity, and morphological uniqueness [5]. Subsequent policy milestones—including the Decision on Strengthening the Protection of Ancient and Notable Trees (1996) and the National Technical Specifications for Ancient and Notable Tree Census and Documentation (2001)—standardized nationwide surveys and defined long-lived trees as those aged 100 years or more [6]. The most recent Regulations on the Protection of Ancient and Notable Trees (2025) further institutionalized legal protection, marking a new stage in the systematic management of long-lived tree heritage.

Although substantial progress has been made in understanding plant senescence and longevity, existing studies have predominantly focused on intrinsic physiological mechanisms and genetic determinants, while comparatively overlooking the integrated effects of external environmental factors. In reality, plant longevity is not solely determined by inherent biological traits but emerges from the combined influence of natural adaptability and anthropogenic contexts. Specifically, plant–environment interactions are complex and multifaceted. First, environmental suitability is strongly species-specific: habitats that support the long-term persistence of certain taxa may not be conducive to others. Second, environmental favorability is inherently relative. Conditions that are generally considered optimal for most plants may, due to subtle variations in light availability, water supply, or soil physicochemical properties, impose constraints on the survival and regeneration of specialized long-lived species. Third, the role of human-mediated environments should not be underestimated. Through practices such as ex situ conservation, religious protection, landscape management, and cultural traditions, humans have created relatively stable habitats for particular plant species. These interventions not only facilitate the preservation of long-lived individuals but may also extend their lifespan beyond natural limits. Therefore, a comprehensive understanding of plant longevity requires integrating intrinsic biological mechanisms with their specific ecological settings and anthropogenic influences. Only by situating longevity within this broader environmental and cultural framework can its underlying nature be more fully and accurately elucidated.

Relevant literature for this review was retrieved from multiple scientific databases, including Web of Science, Scopus, and Google Scholar. Searches were conducted using combinations of keywords such as “tree longevity”, “plant senescence”, “long-lived trees”, “ancient trees”, “secondary metabolites”, “stress resistance”, “tree aging”, “DNA repair”, “telomerase activity”, “anthropogenic influences”, and “tree conservation”. Publications from 1990 to 2025 were primarily considered, with particular emphasis on recent advances in plant physiology, molecular biology, ecology, and conservation science. Both original research articles and review papers were included. In addition, selected policy documents and culturally related studies were incorporated to evaluate anthropogenic influences on long-lived trees. Literature was prioritized based on relevance to tree longevity mechanisms, methodological rigor, and representativeness across different species and ecological contexts.

## 2. Rethinking Senescence and Longevity

### 2.1. Mechanisms of Plant Senescence

Recent perspectives have challenged traditional views that aging in trees inevitably leads to progressive physiological decline. Piovesan and Biondi [7] proposed that tree longevity should be interpreted as a dynamic ecological and evolutionary strategy rather than merely an extension of lifespan. They emphasized that many long-lived trees maintain functional stability through modular growth, continuous meristematic renewal, and adaptive plasticity, thereby partially escaping whole-organism senescence. This perspective suggests that tree longevity is not simply the absence of aging, but rather the outcome of long-term coordination among growth, repair, and environmental adaptation mechanisms. Plant senescence is generally regarded as an inevitable physiological process during the plant life cycle. Extensive studies have investigated senescence mechanisms at multiple organizational levels, leading to the development of various hypotheses. Volkava et al. [8] reported that woody plants, particularly long-lived trees, do not necessarily exhibit pronounced whole-organism senescence. Instead, they may display “negligible senescence,” whereby physiological functions remain relatively stable over extended time scales despite increasing age.

Bond [9], in a comprehensive review, indicated that with increasing tree age and size, some species exhibit declines in photosynthetic rate and stomatal conductance. As trees grow larger, the increased path length for xylem water transport raises hydraulic resistance, thereby reducing water transport efficiency and ultimately constraining leaf photosynthetic capacity. In large trees, structural enlargement may further exacerbate hydraulic limitations, resulting in reduced growth rates.

Jordi et al. [10] demonstrated that during early leaf senescence, chloroplasts shrink, starch granules decrease, chlorophyll content declines, and ribulose-1,5-bisphosphate levels are reduced, leading to decreased electron transport activity and impaired photophosphorylation. In the final stages of senescence, chloroplast integrity is lost and nuclear fragmentation occurs. Wang et al. [11] found that in long-lived *Ginkgo biloba* trees, cambial cell layers decrease and annual ring width narrows with age, accompanied by reduced auxin levels, increased abscisic acid levels, and downregulation of genes related to cell division and differentiation. Trunk cross-sectional growth remains substantial, suggesting that cambial stem cells retain considerable proliferative capacity; no significant age-related decline was observed in photosynthetic traits, reproductive capacity, or senescence-associated gene expression in leaves.

Tian et al. [12] highlighted that reactive oxygen species (ROS) accumulation is a key regulator of plant senescence. During senescence, the balance between ROS production and scavenging is disrupted, leading to progressive oxidative damage when antioxidant systems become insufficient. Wu et al. [13] suggested that under adverse environmental conditions, plants may accelerate senescence to reallocate internal nutrients, thereby enhancing survival and reproductive success. Environmental stresses such as drought, salinity, low temperature, and pathogen infection may induce premature senescence via hormonal signaling pathways. Zhang et al. [14] further emphasized that plant senescence is a complex process regulated by multiple interacting pathways, including phytohormone signaling, environmental cues, and transcription factor networks.

### 2.2. The Dialectical Relationship Between Aging and Longevity

Plant senescence and longevity are not simply opposing phenomena but rather represent a dynamic and evolving balance. From a biological perspective, both are outcomes of the interplay among energy allocation, gene regulation, and environmental adaptation, collectively shaping plant survival strategies across diverse ecological contexts.

Senescence and longevity reflect a dialectical unity between process and outcome. Senescence should not be viewed merely as passive deterioration, but as a highly regulated, genetically programmed process, the core function of which is nutrient remobilization and redistribution. Leaf senescence facilitates the translocation of essential nutrients such as nitrogen and phosphorus to reproductive organs or perennial tissues, thereby ensuring reproductive success and long-term survival. In contrast, longevity can be interpreted as the delay or partial escape from this process. Long-lived plants do not avoid senescence per se; rather, they significantly postpone organismal decline through enhanced self-repair capacity, genome stability, and strong tolerance to environmental stress. In this sense, senescence represents a programmed “exit strategy,” whereas longevity reflects an advanced regulation of this exit process.

In woody plants, this relationship is further characterized by pronounced spatial heterogeneity, manifested as the coordination between local sacrifice and whole-organism persistence. Plants maintain long-term viability by selectively sacrificing specific organs; for example, leaf abscission reduces transpirational water loss, while heartwood formation in xylem limits the spread of pathogens [15]. Critically, meristematic tissues—particularly vascular cambium cells—can retain high proliferative activity and remarkably low mutation rates over centuries in many long-lived species [11]. This sustained “youthfulness” at the cellular level constitutes a fundamental basis for organismal longevity. Thus, orderly local senescence is not detrimental but instead essential for prolonged whole-plant survival. Fojtová et al. [16] emphasized that plant longevity fundamentally differs from animal aging because plants exhibit strong modularity and organ-level turnover. While leaves, flowers, and peripheral tissues undergo continuous senescence and replacement, meristematic regions may retain long-term proliferative potential, allowing the organism as a whole to persist for centuries. This spatial separation between local senescence and organismal continuity represents a central feature of long-lived plants.

From a systems biology perspective, the balance between senescence and longevity is governed by a finely coordinated network of molecular and physiological processes. On the one hand, sequential activation of senescence-associated genes drives cellular disassembly and metabolic decline. On the other hand, longevity-associated defense mechanisms—including heat shock proteins and antioxidant enzyme systems—function to remove damaged proteins and reactive oxygen species [14]. Hormonal balance, endogenous antioxidant capacity, and the accumulation of specific metabolites collectively form feedback regulatory networks that determine whether plants enter rapid senescence or maintain a prolonged functional state. Notably, unlike animals, many long-lived plants possess highly efficient DNA repair systems and sustained telomerase activity [17], enabling the maintenance of genome integrity over centuries and preventing the accumulation of deleterious mutations that would otherwise accelerate aging.

A multidimensional comparison further highlights the distinctions between these two processes. At the biological level, senescence is characterized by nutrient recycling and programmed cellular degradation, whereas longevity reflects sustained physiological function and repair capacity. In terms of phenotypic traits, senescence involves chloroplast degradation, nucleic acid breakdown, and metabolic slowdown, while longevity is associated with persistent cambial activity and the accumulation of secondary metabolites. Regarding environmental responses, senescence is often inducible, with stresses such as drought, low light, and pathogen attack accelerating its onset, whereas longevity is adaptive, relying on enhanced stress resistance mediated by longevity-associated genes. From an evolutionary standpoint, senescence ensures species reproduction—particularly in annual plants with rapid life cycles—while longevity enables plants to occupy stable ecological niches and buffer against long-term environmental fluctuations.

## 3. Intrinsic Mechanisms Underpinning Long-Lived Trees

### 3.1. Nutrient Utilization Strategies Across Tree Ages

In perennial woody plants, increasing age is accompanied by changes in growth rate, organ structure, and resource demand, resulting in dynamic adjustments in nutrient uptake, allocation, and resorption strategies. Zhang et al. [18] reported that in *Metasequoia glyptostroboides* plantations, carbon (C), nitrogen (N), and phosphorus (P) stoichiometry and nutrient resorption efficiency vary significantly across stand ages. Younger stands exhibit higher leaf phosphorus content and resorption efficiency, whereas increasing stand age is associated with elevated C:P and N:P ratios, indicating potential phosphorus limitation in mature stands. Tang et al. [19] found that in woody species across eastern China, more than half of leaf nitrogen and phosphorus can be resorbed prior to leaf abscission. Nitrogen resorption traits are primarily controlled by plant functional groups, whereas phosphorus resorption is more strongly influenced by abiotic site conditions. Deng et al. [20] showed that in *Robinia pseudoacacia* plantations, nutrient resorption efficiency follows a unimodal pattern with stand development, peaking in middle-aged stands and declining in mature stages. This suggests that plants adjust nutrient conservation strategies through ontogeny. Song et al. [21] demonstrated that plants optimize nutrient utilization by coordinating root uptake capacity with leaf nutrient resorption. In *Metasequoia*, stand age positively affects phosphorus resorption efficiency but has little effect on nitrogen resorption. Yuan et al. [22] further showed that leaf age itself strongly influences nutrient dynamics, with younger leaves containing higher nutrient concentrations, while older leaves progressively export nutrients to developing tissues, leading to declining resorption efficiency with leaf aging.

### 3.2. Age-Related Changes in Antioxidant Enzyme Systems

During plant metabolism, reactive oxygen species such as superoxide anions and hydrogen peroxide are continuously produced. Their accumulation during senescence or under stress conditions can cause oxidative damage to cellular membranes, proteins, and nucleic acids [23]. Arena et al. [24] reported that antioxidant enzyme activities vary significantly across developmental stages, with younger leaves generally exhibiting higher activities than senescent leaves in non-long-lived species. Fraga et al. [25] suggested that polyamines play a regulatory role in tree senescence, and total polyamine content may serve as an indicator of aging. Plant senescence is commonly accompanied by oxidative stress, characterized by increased malondialdehyde (MDA) accumulation and altered antioxidant enzyme activities, including superoxide dismutase (SOD), which are widely regarded as physiological indicators of cellular aging and membrane lipid peroxidation [26]. Zhang et al. [27] found that in *Platycladus orientalis*, peroxidase activity decreases significantly with age, while SOD activity in 1200-year-old trees is markedly lower than in younger individuals, and MDA content is significantly higher. In contrast, Turfan et al. [28] reported that in *Castanea sativa*, hydrogen peroxide and MDA contents increase with age, accompanied by elevated antioxidant enzyme activities, suggesting enhanced oxidative stress tolerance in older trees.

### 3.3. Phytohormonal Regulation Across Tree Ages

Plant aging is accompanied by significant changes in endogenous hormone levels and their balance, making phytohormones a critical perspective for studying senescence. Liu et al. [29] showed that the relative proportions of hormones are more indicative of plant developmental status than individual hormone concentrations. Auxin levels are typically higher during juvenile stages, whereas gibberellins and cytokinins are relatively more abundant in mature stages. Studies on *Larix gmelinii* [30] indicate that growth-promoting hormones regulate cambial activity and annual ring formation, thereby influencing growth rate and biomass accumulation. However, endogenous cytokinin levels decline during senescence. Similarly, Gan et al. [31] reported that cytokinin concentrations are significantly higher in young tissues than in senescent ones. Ally et al. [32], using molecular clock approaches, suggested that although old trees remain viable, their sexual reproductive capacity may decline due to accumulated cellular mutations. In contrast, Mencuccini et al. [33] found that grafted branches from old trees exhibit growth rates and seed quality comparable to those of young trees, with no significant differences in hormone or pigment levels, indicating that aging effects may be reversible at the organ level.

Pasques and Munné-Bosch [34] suggested that extreme longevity in mountain pine trees is closely linked to sustained physiological resilience, including efficient antioxidant defense, maintenance of photosynthetic stability, and coordinated hormonal regulation. Their findings indicate that long-lived trees may achieve lifespan extension not through metabolic inactivity, but through long-term maintenance of functional homeostasis under chronic environmental stress. Lu et al. [35] demonstrated that the NAC transcription factor *GbNAC2* enhances salt tolerance in *Ginkgo biloba* through coordinated regulation of flavonoid biosynthesis and hormonal signaling pathways. Specifically, *GbNAC2* directly activates ABA-related signaling while simultaneously promoting flavonoid accumulation, thereby improving antioxidant capacity and mitigating oxidative damage under salinity stress. Importantly, this regulatory mechanism reflects a coordinated balance between growth and defense, suggesting that long-lived trees may achieve longevity partly through dynamic optimization of metabolic investment under stressful conditions. Additional transcriptomic analyses of rejuvenated ancient Ginkgo trees further revealed that flavonoid biosynthesis, phytohormone regulation, and stress-response pathways remain highly active even in extremely old individuals, indicating that long-lived trees may maintain physiological resilience through sustained molecular regulatory capacity rather than simple metabolic slowdown [36].

### 3.4. Secondary Metabolites and Age-Dependent Variation

Secondary metabolites play crucial roles in plant defense against biotic and abiotic stresses and act as key components of signaling pathways [37]. Liu et al. [38] pointed out that many long-lived tree species exhibit an expansion of gene families related to secondary metabolite synthesis at the genomic level, which can promote the synthesis of terpenes, phenolics, and other defensive metabolites, thereby enhancing the trees’ ability to withstand environmental stress and pests, providing genomic-level evidence for the role of secondary metabolites in maintaining the long-term survival of trees. Yan et al. [39] reported that age significantly influences the synthesis and accumulation of secondary metabolites, with older plants exhibiting higher levels of phenolic and lipid compounds. Age-dependent metabolic reprogramming enhances stress resistance by increasing both primary and secondary metabolism. Shi et al. [40] found that the contents of phenolic acids and flavonoids in leaves change significantly with age, with catechin compounds generally increasing. These changes are associated with altered expression of key genes in the phenylpropanoid pathway. Metabolomic studies in Andean plants [41] further indicate that metabolite diversity and abundance increase with plant age, enhancing adaptability to complex environments. Cui et al. [42] suggested that secondary metabolites play a particularly important role in long-lived tree species. Many such species exhibit expansion of gene families related to secondary metabolite biosynthesis, facilitating the production of terpenoids, phenolics, and other defense compounds, thereby improving resistance to environmental stress and pathogens.

### 3.5. Age-Related Changes in Metabolic Pathways and Functional Genes

Advances in molecular biology, particularly high-throughput sequencing technologies, have enabled detailed investigation of age-related molecular mechanisms in plants. Numerous genes associated with tree aging have been identified. Transcriptomic analyses of *Cunninghamia lanceolata* [43] revealed that tree age significantly affects the expression of genes involved in cell wall formation and carbon metabolism. Genes related to cellulose synthesis and secondary wall formation show distinct age-dependent expression patterns, closely linked to wood formation. Genes involved in starch and sugar metabolism also exhibit significant variation across developmental stages, indicating that carbon metabolic pathways are tightly regulated by age. Transcription factor families involved in cell differentiation, secondary wall formation, and stress responses display stage-specific expression patterns. Studies on long-lived cypress trees [44] identified multiple functional proteins involved in polyamine metabolism, defense, and ROS scavenging under low-temperature stress. Li et al. [45] demonstrated that tree aging involves extensive transcriptomic reprogramming, with thousands of differentially expressed genes enriched in processes such as cell differentiation, hormone signaling, and xylem formation. The *miR156–SPL* regulatory network has been identified as a central component of the plant aging pathway [46], with high expression in juvenile stages and gradual decline with age, thereby promoting the transition to maturity. Long-term transcriptomic monitoring by Lu et al. [47] revealed that gene expression variability is greater during growth stages than during senescence. Nutrient remobilization-related transporter genes are activated during late senescence, facilitating nutrient translocation, while RNA splicing factors and photoperiod-responsive regulators fine-tune the senescence process.

### 3.6. Genomic Stability and DNA Maintenance in Long-Lived Trees

Genomic stability is increasingly recognized as a fundamental prerequisite for extreme longevity in trees. Unlike short-lived plants, long-lived tree species must maintain genome integrity over decades or even centuries despite continuous exposure to environmental stress, ultraviolet radiation, oxidative damage, and replication-associated mutations. Popov et al. [48] further proposed that plant longevity is strongly associated with conserved genetic mechanisms regulating genome maintenance, oxidative stress responses, hormonal signaling, and epigenetic modification. Unlike many animals, plants possess highly flexible developmental programs and continuous meristematic activity, enabling long-term replacement of damaged tissues and dynamic adaptation to environmental stress. The authors emphasized that understanding aging in plants requires integrating genetic stability with ecological and developmental plasticity. Unlike many animals, plants often retain telomerase activity in actively dividing tissues throughout much of their lifespan, particularly within meristems. Sustained telomerase activity may help prevent excessive telomere shortening and preserve chromosomal stability during prolonged growth. Recent reviews on the tertiary relict tree *Eucommia ulmoides* have emphasized that long-lived woody plants often exhibit extensive accumulation of bioactive compounds together with complex genetic regulatory systems involved in stress adaptation and secondary metabolism. These traits may jointly enhance environmental tolerance and contribute to long-term persistence [49]. Batalova and Krutovsky [50] highlighted that epigenetic regulation may constitute an important mechanism underlying tree longevity. DNA methylation dynamics, chromatin remodeling, and stress-induced epigenetic memory potentially enable long-lived trees to maintain genomic stability and adaptive flexibility over extended time scales. The authors further proposed that epigenetic modifications may mediate long-term responses to environmental stress without requiring permanent genetic changes.

## 4. Environmental Drivers of Long-Lived Trees

The longevity of long-lived trees is fundamentally shaped by their biophysical environment. Key abiotic drivers include latitude, altitude, climatic variability, edaphic properties, and geomorphological context, all of which jointly regulate resource availability and physiological stress [51]. Recent large-scale comparative analyses further demonstrate that different evolutionary lineages may achieve longevity through contrasting strategies. Brienen et al. [52] reported that gymnosperms and angiosperms exhibit distinct pathways toward extended lifespan. Gymnosperms generally rely on slow growth, dense wood structure, and conservative resource-use strategies, whereas angiosperms more frequently achieve longevity through efficient hydraulic regulation and adaptive plasticity. These findings suggest that tree longevity is not governed by a universal mechanism but rather arises through multiple evolutionary trajectories shaped by ecological context and life-history trade-offs.

Latitude and longitude not only determine the spatial distribution of moisture and heat but also shape major biogeographical patterns. Comparative studies show that tropical tree species, although growing at approximately twice the rate of temperate and boreal species, exhibit substantially shorter lifespans. The estimated mean lifespan of tropical trees is 186 ± 138 years, whereas temperate species exhibit an average lifespan of 322 ± 200 years, reflecting a clear latitudinal gradient in tree longevity [53].

Elevation creates microclimatic mosaics that influence growth and senescence dynamics through shifts in temperature, precipitation, radiation, and soil fertility. Both empirical and modeling studies indicate that tree diversity and biomass show unimodal relationships with altitude, while human disturbance generally diminishes at higher elevations. In harsher alpine or subalpine habitats, slower growth and reduced biotic pressure can promote exceptional longevity. For example, *Fagus sylvatica* commonly survives ~300 years in fertile lowlands but may exceed 600 years under nutrient-poor, high-elevation conditions [54]. Similarly, long-lived tree assemblages on the Qinghai–Tibet Plateau persist largely due to minimal anthropogenic interference despite extreme altitude.

Local habitat conditions further play a critical role in determining the persistence of long-lived trees. As trees age, their growth vigor declines, while surrounding younger individuals generally display high competitiveness. Intense competition for resources results in insufficient light, poor ventilation, and elevated humidity, conditions that impair tree health and promote the proliferation of pathogenic organisms. Encroachment by neighboring saplings progressively reduces the available growing space, ultimately leading to diminished competitiveness, canopy suppression, and eventual mortality. Endophytic microorganisms are increasingly recognized as key contributors to the survival and longevity of long-lived trees. These endophytes reside within plant tissues without causing disease and can enhance host resistance to environmental stresses. A study on *Platanus orientalis* in central Iran reported that endophyte frequency was significantly higher in long-lived trees than in juvenile individuals, and endophyte presence was positively correlated with leaf nutrient content, tree height, and trunk circumference [55]. Symbiotic interactions between fungi and endophytes promote nutrient assimilation and contribute to improved physiological performance in older trees. Endophytic fungi benefit from host-derived nutrients and protection, while simultaneously enhancing host nutrient uptake and stress tolerance. Thus, maintaining or inoculating beneficial endophytes may substantially increase the survival and resilience of long-lived trees, particularly under harsh environmental conditions.

Mechanical and climatic stresses impose additional constraints on long-lived trees. As trees increase in height and crown size, the mechanical load on their roots and stems intensifies, rendering them more vulnerable to windthrow or trunk breakage. Studies indicate a strong positive correlation between tree height and susceptibility to wind damage [56]. For evergreen conifers, accumulation of heavy snow on large crowns may cause severe crown deformation or collapse. In addition, tall trees experience higher exposure to lightning strikes; quantitative analyses in the forests of Panama identify lightning as a major driver of tree mortality. On average, each lightning strike directly kills approximately 3.5 trees and damages over 11.4 surrounding individuals.

## 5. Anthropogenic Influences on Long-Lived Trees

Beyond environmental constraints, the endurance of long-lived trees is deeply intertwined with human culture and social institutions. Across civilizations, these organisms have been revered as symbols of vitality, wisdom, and moral virtue, shaping collective attitudes toward nature and fostering long-term protection [57]. The historical dimension represents a core attribute of long-lived tree culture, as historicity is inherently linked to temporal longevity. According to classification and grading standards, increasing age corresponds to stronger historical value. Long-lived trees are frequently regarded as “living cultural relics,” “living fossils,” and “living archives,” serving as unique biological records for research across disciplines such as ecology, history, sociology, and anthropology. Through their extended lifespans, long-lived trees document long-term environmental changes, embody collective memory, and reflect the continuity of regional cultural development.

Anthropogenic protection of long-lived trees does not follow a single static model but instead represents a progressive continuum ranging from culturally mediated passive protection to highly intentional technological intervention. This continuum can be differentiated according to the intensity of management intention, technological dependence, and ecological intervention. At the earliest stage, long-lived trees are protected through sacred beliefs, ancestral worship, or geomantic traditions that restrict disturbance and exploitation. This form of protection is fundamentally prohibitive, relying on cultural taboos and social norms rather than direct ecological management. Although effective in reducing human disturbance, such protection has limited capacity to mitigate physiological decline in aging trees. Church forests in Ethiopia, sacred groves in Rome, and cemetery woodlands in Germany all demonstrate the protective power of religious and cultural norms [58]. Many temples and shrines were built around preexisting long-lived trees, forming enduring “temple–tree symbioses” that integrate ecology with faith [59].

As cultural restrictions become institutionalized, conservation shifts toward community-based governance characterized by explicit regulations, land-use restrictions, and social enforcement mechanisms. This stage represents a transition from symbolic protection to organized environmental governance, although interventions still primarily function by limiting external disturbance rather than directly regulating tree physiology. Studies on sacred groves in India have shown that traditional religious taboos gradually evolved into community-based governance systems characterized by land-use restrictions, collective supervision, and social sanction mechanisms. Such systems effectively reduced anthropogenic disturbance and contributed to the long-term persistence of culturally protected forests and ancient trees, although their stability increasingly depends on the continuity of local cultural institutions under modernization pressures [60].

With the development of modern conservation systems, long-lived trees increasingly become integrated into formal landscape and spatial management frameworks through protected zones, fencing, visitor regulation, and habitat planning. Conservation at this stage emphasizes spatial isolation and disturbance control while incorporating ecological and geographical management strategies. Modern protected-area management increasingly relies on zoning systems, buffer areas, visitor regulation, and landscape-scale planning to reduce anthropogenic disturbance while maintaining ecological connectivity and long-term ecosystem stability. Such approaches reflect a transition from isolated protection toward integrated spatial governance combining ecological conservation with land-use management and human activity regulation [61].

More direct intervention occurs through routine arboricultural practices such as irrigation, fertilization, pruning, pest management, and soil improvement. Here, conservation objectives shift from passive preservation to active maintenance aimed at sustaining physiological vitality and delaying senescence processes. Active management practices, including pruning, structural stabilization, pest control, and rhizosphere improvement, can enhance physiological resilience and reduce mechanical failure risks in aging trees. Such interventions are increasingly viewed not merely as maintenance measures, but as adaptive strategies for prolonging tree vitality, sustaining habitat functions, and improving ecosystem resilience in managed landscapes [62].

At the highest intervention level, conservation becomes restorative and biotechnology-oriented. Intensive measures—including structural support, cavity repair, root rejuvenation, grafting, tissue culture, and ex situ genetic conservation—are employed to mitigate severe physiological or structural decline. This stage reflects a fundamental transition in conservation philosophy, from preventing destruction to actively prolonging lifespan and maintaining long-term biological continuity in long-lived trees. Advanced conservation strategies increasingly rely on cryobiotechnology, tissue culture, grafting, and ex situ germplasm preservation to maintain the long-term viability of threatened tree species, particularly those producing recalcitrant seeds that cannot be conserved through conventional seed banking. Such approaches reflect a shift from passive protection toward restoration-oriented biological conservation aimed at preserving both physiological vitality and genetic continuity in long-lived trees [63].

## 6. Coupling of Intrinsic and Extrinsic Factors

The preceding analysis indicates that plant longevity is neither a purely genetically predetermined trait nor a linear outcome of single environmental drivers. Rather, it emerges from the interactive effects of intrinsic biological mechanisms, natural environmental pressures, and anthropogenic regulation. To move beyond traditional approaches that treat intrinsic and extrinsic factors in isolation, it is necessary to establish an integrative theoretical framework in which plant longevity is conceptualized as a multidimensional, nonlinear, and dynamically evolving coupled system. Here, we propose a four-dimensional coupling model—comprising resource allocation, stress resistance, environmental pressure, and anthropogenic regulation—to explain how plants achieve delayed senescence and extended lifespan within complex systems.

### 6.1. Conceptual Structure of the Four-Dimensional Coupling Model

Recent integrative perspectives have further emphasized that tree longevity cannot be explained solely by isolated molecular or environmental factors. Liu et al. [38] proposed that longevity emerges from multifaceted interactions among genetic regulation, metabolic coordination, stress adaptation, and ecological context. Their framework supports the view that long-lived trees should be interpreted as dynamically regulated systems in which physiological resilience, environmental filtering, and developmental plasticity collectively shape lifespan potential.

The central premise of this model is that plant longevity arises from continuous interactions and energy–information exchanges among four key subsystems. Perturbations in any single subsystem can propagate through coupling relationships and induce system-wide adaptive responses. Resource allocation constitutes the intrinsic driving axis of the model. Throughout development, plants face trade-offs among growth, defense, and maintenance. Longevity fundamentally depends on preferential allocation of limited resources toward maintenance metabolism and defensive functions. Sustained accumulation of secondary metabolites, energetic support for DNA repair systems, and substrate supply for cambial cell division all rely on optimized allocation strategies. This subsystem defines the internal “metabolic budget” that underpins longevity potential (Table 1).

Stress resistance represents the intrinsic defense axis, encompassing the plant’s capacity to prevent, tolerate, or repair damage at molecular, cellular, and organismal levels. It includes antioxidant enzyme activity, heat shock protein expression, cell wall mechanical strength, and the efficiency of symbiotic networks such as mycorrhizae. Importantly, stress resistance is not static but dynamically regulated by resource allocation. Increased investment in defense enhances resistance thresholds, whereas excessive allocation to growth or reproduction may compromise defensive capacity.

Environmental pressure functions as the external filtering axis, including climatic variability, soil physicochemical conditions, hydrological regimes, biotic competition, and pathogen stress. Environmental pressure exerts dual effects on plant longevity. Moderate stress may enhance adaptive capacity through mechanisms such as systemic acquired resistance and epigenetic modification, whereas excessive or prolonged stress can cause irreversible damage and accelerate senescence. Thus, environmental pressure serves as a critical test of whether internal resource allocation and resistance capacity are sufficient to sustain longevity.

Anthropogenic regulation constitutes the human-mediated intervention axis, reflecting deliberate modifications of plant environments through cultural practices, management techniques, and spatial planning. Unlike the other subsystems, anthropogenic regulation is highly goal-oriented and adjustable. It can reduce environmental stress, optimize resource availability, and prevent disturbances through cultural or legal protection. Functionally, it acts as an external regulator that modulates the coupling strength and equilibrium among the other three subsystems.

### 6.2. Dynamic Properties of the Coupled System: Feedback, Resilience, and Stability

The four subsystems form an integrated whole through complex coupling mechanisms. Their dynamics can be characterized by three key properties: feedback, resilience, and stability. Feedback mechanisms are central to system self-regulation. Two primary types of feedback loops can be identified. First, negative feedback loops promote system stabilization. For example, when environmental stress increases and induces physiological damage, timely anthropogenic intervention can reduce defense demands, allowing reallocation of resources toward repair and maintenance, thereby slowing senescence and restoring system equilibrium. Second, positive feedback loops may lead to system destabilization. Persistent allocation toward defense at the expense of growth, or maladaptive management practices, can reduce physiological efficiency, weaken stress resistance, and increase vulnerability to environmental pressures. This may generate a reinforcing cycle of “accelerated senescence–reduced resistance–damage accumulation.” Understanding the directionality and threshold behavior of these feedbacks is essential for predicting longevity trajectories.

Resilience describes the capacity of the system to recover from disturbances and return to its original functional state or transition to a new stable configuration. In long-lived plants, resilience is reflected in the ability to repair and regenerate following extreme climatic events, pest outbreaks, or physical damage. High resilience depends on intrinsic resource redundancy and defensive reserves and effective anthropogenic support that provides temporal windows for recovery. However, resilience is finite; repeated or intense disturbances may exhaust recovery capacity, shifting the system from reversible perturbations to irreversible structural decline.

Stability refers to the system’s ability to maintain longevity over extended time scales through dynamic equilibrium. It emerges from the coordinated balance among resource allocation efficiency, stress resistance capacity, environmental pressure intensity, and anthropogenic regulation. Stability does not imply stasis but rather sustained balance among competing processes, including senescence and repair, stress and recovery, and resource consumption and replenishment. Highly stable systems are characterized by effective buffering of environmental fluctuations, flexible adjustment of allocation strategies across developmental stages, and timely human intervention as an external stabilizer. Conversely, abrupt changes in any subsystem—such as rapid climate shifts or disruption of management practices—can destabilize the system and compromise longevity.

### 6.3. Theoretical Implications and Practical Applications

The primary theoretical contribution of this framework lies in moving beyond reductionist paradigms of genetic or environmental determinism and emphasizing the nonlinear, interactive nature of plant longevity. Within this perspective, longevity is not simply the outcome of an optimal genotype in an ideal environment but an emergent property arising from continuous interactions among intrinsic potential, external constraints, and human intervention.

From an applied perspective, the model provides a systematic basis for the conservation and management of long-lived trees. Effective strategies should not rely on single interventions but instead assess the position of individual trees within the four-dimensional system. For example, trees experiencing high environmental stress, low stress resistance, and insufficient management are likely near critical thresholds and require integrated interventions. In contrast, under moderate stress and sufficient internal resource reserves, excessive human intervention may disrupt natural feedbacks and reduce resilience. Furthermore, this framework offers a conceptual tool for predicting plant longevity under global change by simulating how shifts in environmental pressure and management regimes affect system dynamics, thereby enabling assessment of species vulnerability and adaptive potential.

## 7. Conclusions and Future Perspectives

There is an old Chinese saying: “Opportune time, advantageous location, and harmony among people.” The formation of ancient trees can actually be perfectly summarized by this saying. The exceptional longevity of long-lived trees is not an isolated biological phenomenon but rather the outcome of synergistic interactions among intrinsic genetic programs, natural environmental selection, and anthropogenic influences. Senescence, as a programmed and actively regulated process, provides an evolutionary foundation for nutrient recycling and reproductive assurance [64]. In contrast, longevity represents the extended regulation and temporal modulation of this program, relying not only on sustained meristematic activity and efficient DNA repair systems but also on habitat suitability and human management. Despite substantial advances in elucidating molecular mechanisms and in case-based conservation practices, current research paradigms still exhibit critical gaps that both constrain understanding and point toward future directions.

### 7.1. Major Limitations in Current Research

Lack of systematic long-term monitoring data. Tree longevity unfolds over decades to centuries, yet most existing studies are based on short-term experiments or cross-sectional surveys, limiting the ability to capture dynamic trajectories of senescence and persistence over time. For example, continuous evidence remains scarce regarding post-disturbance recovery following extreme climatic events, threshold effects of chronic pathogen accumulation, and long-term trends in secondary metabolite dynamics across ontogeny. This temporal discontinuity restricts many conclusions to correlative interpretations and hinders the development of predictive, causally grounded models of plant longevity.

Disconnection between molecular mechanisms and ecological scales. Current research often exhibits a polarized structure: one end focuses on gene expression and signaling pathways under controlled laboratory conditions, while the other relies on descriptive field-based ecological observations. Critical questions remain unresolved: How do molecular regulatory networks of senescence respond to habitat heterogeneity? Do epigenetic modifications mediate long-term “memory” of environmental stress? Can human management practices induce sustained activation of longevity-associated defense pathways at the molecular level? In essence, integrative, multi-scale research spanning genes, individuals, populations, ecosystems, and anthropogenic landscapes remains insufficient, thereby limiting the translation of fundamental discoveries into effective conservation strategies.

### 7.2. Key Directions for Future Research

Advancing multi-omics integration and cross-scale linkage. Future studies should move beyond single-omics approaches by integrating genomics, transcriptomics, proteomics, metabolomics, and epigenomics to construct a comprehensive molecular landscape of plant longevity. Crucially, these datasets need to be coupled with long-term in situ monitoring of physiological traits, microclimatic variables, and soil microbiomes to establish “molecule–physiology–environment” association frameworks. By resolving how longevity-associated gene expression varies across environmental gradients, it will be possible to distinguish conserved molecular modules from environment-dependent ones, thereby providing biomarkers and theoretical foundations for predicting longevity potential under diverse ecological conditions.

Quantifying human intervention mechanisms and developing operational models. Although the positive role of anthropogenic environments in promoting plant longevity is widely acknowledged, the underlying mechanisms remain largely qualitative. Future research should employ controlled experiments and long-term comparative studies to quantify the contributions of specific management practices to physiological performance, structural stability, and lifespan extension of long-lived trees. In parallel, comprehensive evaluation systems incorporating cultural value, cost–benefit efficiency of conservation efforts, and ecosystem service outcomes should be developed. Such frameworks would elevate human factors from background variables to core explanatory drivers, thereby enabling evidence-based decision-making in conservation practice.

Reassessing plant longevity under global change scenarios. Global change drivers—including climate warming, land-use transformation, and biological invasions—are fundamentally reshaping plant habitats, potentially transforming historically suitable environments into stress-prone systems. Future research should address how increasing frequencies of extreme climatic events disrupt physiological homeostasis in long-lived plants, whether elevated atmospheric CO_2_ and rising temperatures alter the thresholds for senescence initiation, and to what extent human interventions can buffer negative impacts on long-lived tree populations. Developing predictive models of plant longevity under climate change scenarios will be essential for assessing species vulnerability, identifying adaptive potential, and informing forward-looking conservation strategies.

## Figures and Tables

**Table 1:** Potential measurable indicators for the four-dimensional coupling framework of tree longevity.

Coupling Dimension	Functional Meaning	Candidate Measurable Indicators	Representative Examples
Resource allocation	Coordination among growth, maintenance, and defense	C:N:P stoichiometry, nutrient resorption efficiency, non-structural carbohydrate content, biomass allocation	Leaf N:P ratio, phosphorus resorption efficiency
Stress resistance	Capacity to tolerate and repair physiological damage	SOD, POD, CAT activities, MDA content, flavonoid accumulation, heat shock protein expression	Antioxidant enzyme activity, ROS scavenging capacity
Environmental pressure	External abiotic and biotic constraints affecting survival	Drought frequency, temperature variability, soil nutrient availability, pathogen pressure, competition intensity	Hydraulic limitation, climatic extremes
Anthropogenic regulation	Human-mediated modification of habitats and disturbance regimes	Protection status, management intensity, pruning frequency, soil amendment, cultural preservation	Sacred groves, support systems, conservation policies

## Data Availability

No new data were created, or data are unavailable due to privacy or ethical restrictions.
